# Supplementary material for: Accumulation of copy-back viral genomes during respiratory syncytial virus infection is preceded by diversification of the copy-back viral genome population followed by selection
Source: Virus Evol. 2022 Sep 28;8(2):veac091. doi: 10.1093/ve/veac091 (PMC9615430; doi:10.1093/ve/veac091)
Supplement: veac091_Supp [file veac091_supp.zip › suppl_data/Supplementary Data.docx]

**Supplementary Data**

**
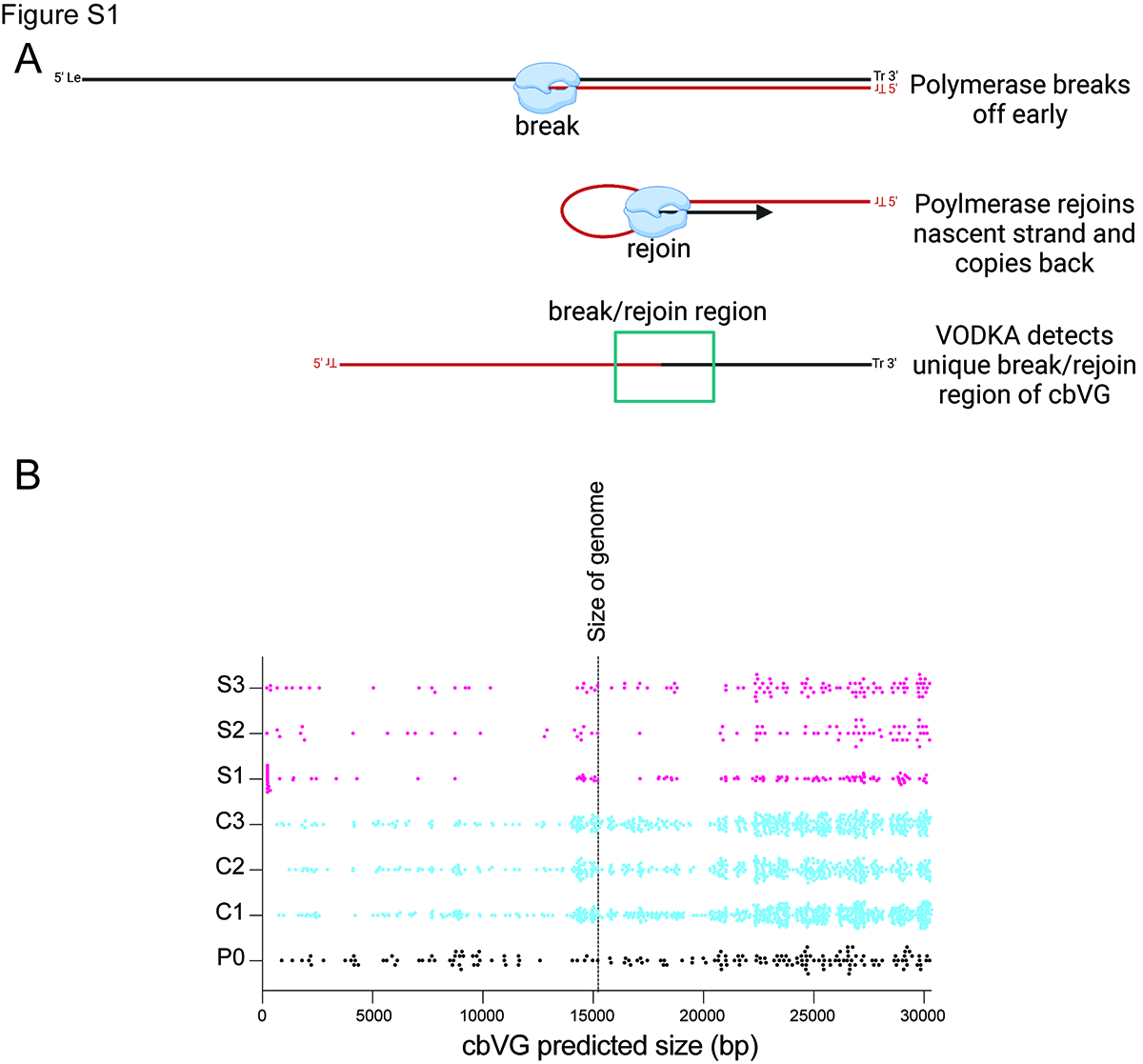
**

Figure S1: RNAseq/VODKA2 approach and distribution of predicted cbVG sizes. (A) Schematic of cbVG generation and cbVG detection by VODKA2. (B) Data represents cbVG predicted size distribution for P0, P20 C1-C3 and P20 S1-S3. Each dot represents a cbVG species. The black dashed line indicates the size of the genome (15223bp).

**
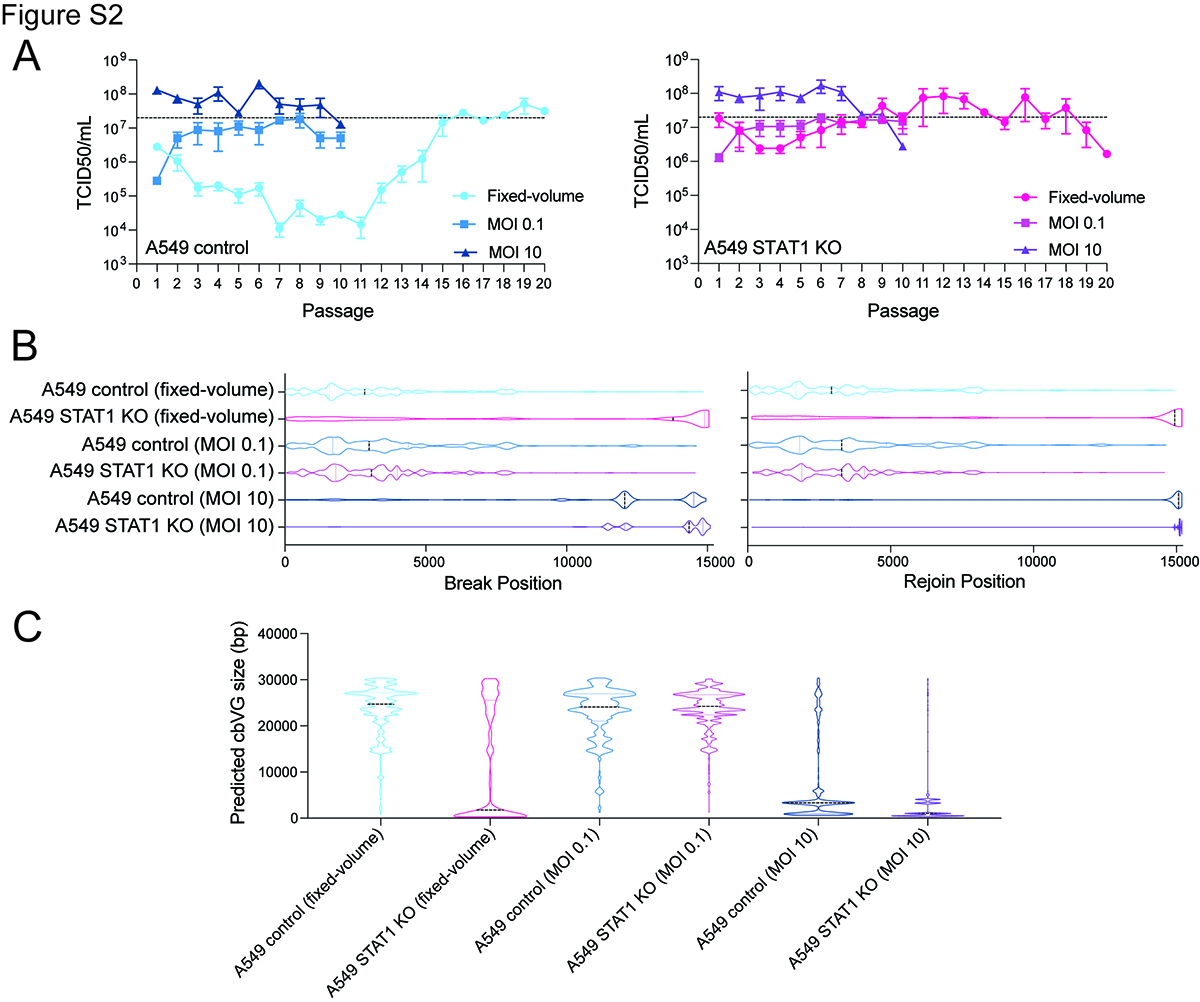
**

Figure S2: Comparison of virus kinetics and cbVG populations between fixed-volume and fixed-MOI experiments. (A) TCID50 assay was performed on Hep2 cells for all samples (n= 3 lineages). Dashed lines indicate 2x10^7 TCID50/ml, which is highest titer MOI 0.1 reached. (B) Graphs represent distribution of break and rejoin positions. Data are shown as truncated violin plots with median and interquartile ranges (n= 3 lineages). (C) Graphs represent distribution of predicted sizes of cbVGs. Data are shown as truncated violin plots with median and interquartile ranges (n= 3 lineages).

**
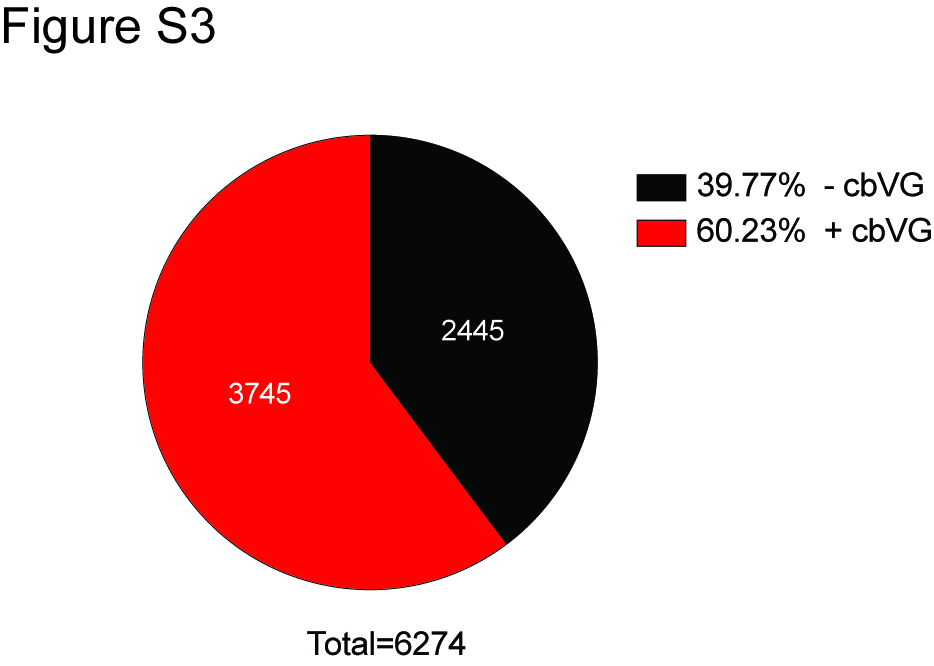
**

Figure S3: Proportions of genomic and antigenomic cbVGs cbVGs from fixed-volume experiment (P0, P20 C1-C3 and P20 S1-S3) were aligned to reference genome to assess strandness. Pie chart indicates proportion of genomic (-cbVG) and antigenomic (+cbVG) cbVGs.

**
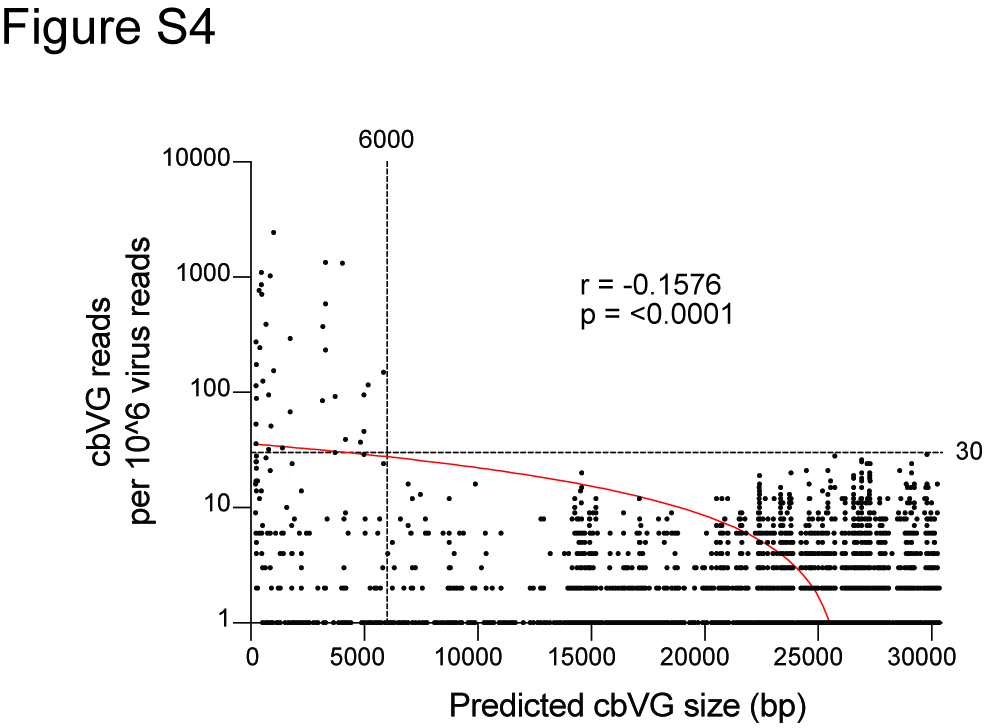
**

Figure S4: Correlation between predicted cbVG size and cbVG reads. Each dot represents a cbVG species from P20 fixed-volume experiment (C1-C3 and S1-S3) and from P10 fixed-MOI 0.1 and 10 experiments (C1-C3 and S1-S3). P value is shown for Pearson correlation between predicted cbVG size and cbVG reads are indicated. Line of best fit for simple linear regression is shown in red. Black dashed lines indicate 30 reads and 6000 bp.

**
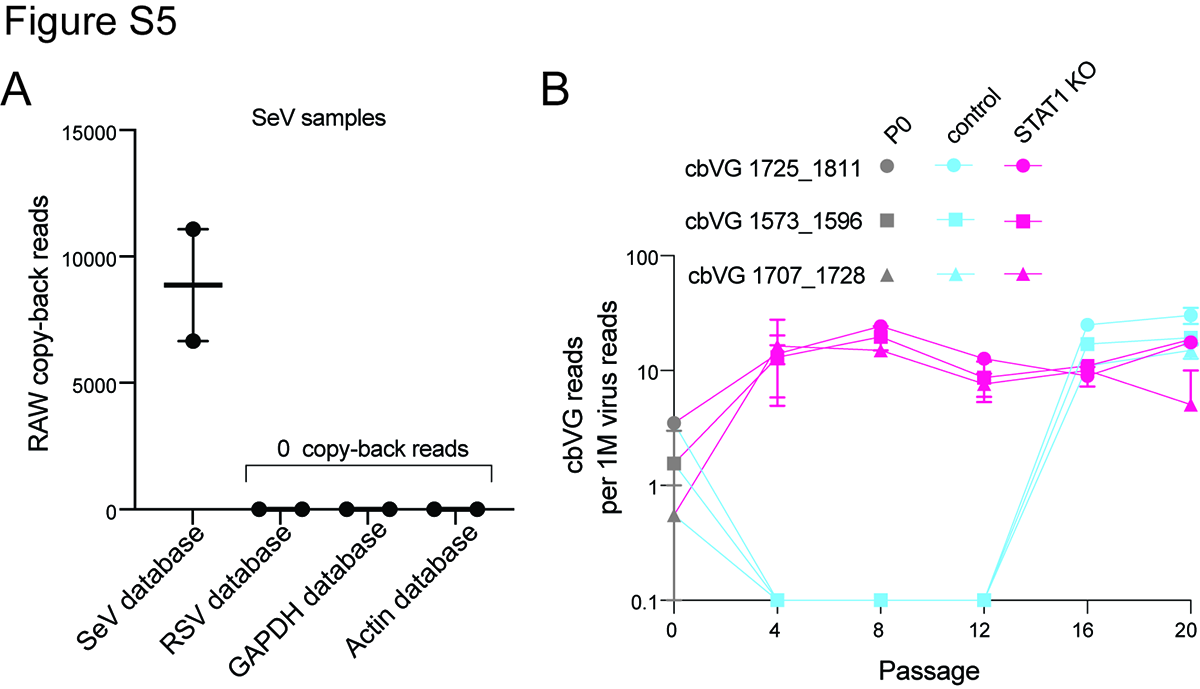
**

Figure S5: Confirmation and tracking of predicted long cbVG species. (A) Two SeV samples from a previous study (Sun et al., 2019; Xu et al., 2017) were analyzed by VODKA2 with an SeV, RSV, GAPDH or Actin database. Raw data are shown as mean ± s.e.m. (n= 2) (B) Line graph shows number of reads for 3 different cbVG species normalized per 1 million virus reads for different passages in A549 control (blue) or STAT1 KO (pink) cells (n= 3 lineages). The break and rejoin positions are indicated in the legend.
